# Profile of patients consulting in child psychiatry for trichotillomania

**DOI:** 10.1192/j.eurpsy.2021.1116

**Published:** 2021-08-13

**Authors:** M. Sahnoun, A. Guedria, N. Gaddour

**Affiliations:** Child Psychiatry Unit, Department Of Psychiatry, Hôpital Fattouma Bourguiba Monastir, monastir, Tunisia

**Keywords:** trichotillomania, Antidepressants, trichophagia, CBT

## Abstract

**Introduction:**

Trichotillomania is a disorder characterized by the compulsive pulling out of one’s own hair. It usually starts just before or after puberty, and about 1 to 2% of people have this disorder. But its incidence is variable over the years and socio-demographic data.

**Objectives:**

Describe the profile of children and adolescents consulting for trichotillomania.

**Methods:**

An incidence survey was carried out among children and adolescents followed for trichotillomania at the out-patient unit of child psychiatry (Monastir - Tunisia) from January 2003 to September 2020.

**Results:**

Among the 11000 patients who were followed during the study period, 47 patients presented trichotillomania, corresponding to a rate of 0.42%. Three of them presented with associated trichophagia and two were operated on in pediatric surgery for trichobezoar. A female predominance was noted with a sex-ratio of 0.37. The average age was 9.3 years with extremes ranging from 2 to 15 years. Almost all of the patients were in school. Most of the patients were referred by dermatologists. We retained in these patients: 17% presented an attachment disorder, 14.8% had a depressive disorder, 6.3% had anxiety elements, 6.3% had an intellectual disability, 4.2% had an associated enuresis and one case had a GAD. The treatment was to undergo behavioral measurements or CBT in 91.4%. Pharmacological management was carried out in 46.8% of patients and was mainly based on antidepressants.

**Conclusions:**

Trichotillomania is a disorder that can be stressful for patients as well as their families. Better knowledge of the profile of these patients is necessary in order to better therapeutic efficacy.

